# Research on the Transmission Characteristics of Air-Coupled Ultrasound in Double-Layered Bonded Structures

**DOI:** 10.3390/ma11020310

**Published:** 2018-02-21

**Authors:** Xing-Guo Wang, Wen-Lin Wu, Zhi-Cheng Huang, Jun-Jie Chang, Nan-Xing Wu

**Affiliations:** 1 School of Mechanical and Electronic Engineering, Jingdezhen Ceramic Institute, Jingdezhen 333403, China; wuwenlin123@126.com (W.-L.W.); huangzhicheng@jci.edu.cn (Z.-C.H.); wunanxing@jci.edu.cn (N.-X.W.); 2 Key Lab of Nondestructive Testing, Ministry of Education, Nanchang Hangkong University, Nanchang 330063, China; chang@jp-probe.com; 3 Japan Probe, 1-1-14 Nakamura Chou Minami Ward Yokohama City, Kanagawa Prefecture 2320033, Japan

**Keywords:** weak bonded, air-coupled ultrasound, transmission peaks, interfacial stiffness, double-layered bonded structures

## Abstract

The ultrasonic transmission spectrum in a double-layered bonded structure is related closely to its interfacial stiffness. Consequently, researching the regularity of the transmission spectrum is of significant interest in evaluating the integrity of the bonded structure. Based on the spring model and the potential function theory, a theoretical model is developed by the transfer matrix method to predict the transmission spectrum in a double-layered bonded structure. Some shift rules of the transmission peaks are obtained by numerical calculation of this model with different substrates. The results show that the resonant transmission peaks move towards a higher frequency with the increase of the normal interfacial stiffness, and each of them has different movement distances with the increasing interfacial stiffness. Indeed, it is also observed that the movement starting points of these peaks are at the specific frequency at which the thickness of either substrate plate equals an integral multiple of half a wavelength. The results from measuring the bonding specimens, which have different interfacial properties and different substrates in this experiment, are utilized to verify the theoretical analysis. Though the theory of “starting points” is not demonstrated effectively, the shift direction and distance exactly match with the result from the theoretical algorithm.

## 1. Introduction

Adhesive bonding has excellent shock absorption capacity and reliable sealing performance in comparison with welding and bolting [1,2]. Since the 1940s, the technique of adhesive bonding has been widely used in automotive, aviation, and spaceflight fields [3], such as the engine cover plate of the automobiles, the envelope of airplanes, and hatches of satellite. If adhesive bonding cannot meet the design requirement or bonding layer aging appears, it may cause some serious accidents [4]. Therefore, how to detect bonding defects, such as voids, inclusions, and weak bonds, etc., is of crucial significance for the prospect of bond technology. 

Compared with the traditional destructive testing method, non-destructive testing (NDT) techniques, which include laser ultrasonic systems, eddy current methods, infrared thermography methods, ultrasound methods, and so on, have been used to evaluate the characteristics of tested materials. Furthermore, NDT techniques do not destroy their intrinsic structure [5,6,7,8]. However, so far every NDT technique has its own advantages and disadvantages [9,10,11,12]. Ultrasonic methods, which have some advantages like high precision, convenient operation, and low cost, are regarded as the most promising techniques to measure the integrity of bonding structures. Thus, it is of great practical interest to research the transmission characteristic of ultrasound propagating in the bonding structures [13,14,15].

The ultrasonic techniques which were used to evaluate the bonding quality have developed very rapidly in the past several years [16,17,18]. Especially, a significant amount of effort has been implemented to demonstrate the correlation between the transmission coefficient and the adhesive bond quality by some researchers [19,20]. Thomson established a theoretical model of ultrasonic wave propagation in multi-layered structures by the transfer matrix method and used this model to calculate the transmission coefficient [21]. Wang et al. studied the ultrasonic transmission on anisotropic media with rigid and slip interfaces emphatically [22]. Their studies focus on the traditional ultrasonic coupling method, however, few attempts have been made to research the transmission characteristics of an air-coupled ultrasonic technique. 

The air-coupled ultrasonic technique has drawn more and more attention in recent years, which is attributed to its advantages [23]. First, this technique does not pollute the tested material surface and wear the probe, and the tested materials could not be damaged by immersing the liquid. Moreover, in light of the convenient installation and high testing speed, this technique has been considered to have the potential for evaluating the bonded quality via on-line monitoring. Some researchers focus on the application of air-coupled ultrasound to assess the lack of adhesive in glued solid wood objects [24,25], and the investigation of the accuracy of ultrasonic air-coupled imaging of defects in composite materials [26]. However, the air-coupled ultrasound cannot completely replace traditional ultrasonic methods (contact and immersion ultrasound) because of its intrinsic drawbacks, for instance, its narrow bandwidth, low signal-to-noise ratio, and poor resolution [23,27]. The reason is that sound waves are well suited to generation in water or, especially, in solids. In air, however, just the opposite is required. Air is very compliant, so waves from a high impedance source couple poorly into the air [28]. Furthermore, it is only suitable for using low frequencies since the ultrasonic attenuation in air sharply increases with the increase of its frequency [29].

It is helpful for the assessment of bonding quality to predict the through-transmission spectrum change with the strength of adhesive bonds. In order to reduce the effects of air-ultrasonic drawbacks on detecting the integrity of adhesive bonds, in this paper, we investigate the laws that resonant peaks in the transmission coefficients shift with the increase of the interfacial stiffness in different substrate materials and thicknesses. These laws provide the theoretical basis for optimizing the detecting parameter. In consideration of theoretical analyses, the change laws have been presented, and also demonstrated by experiments.

## 2. Theory

### 2.1. Physical Model

**Figure 1:**
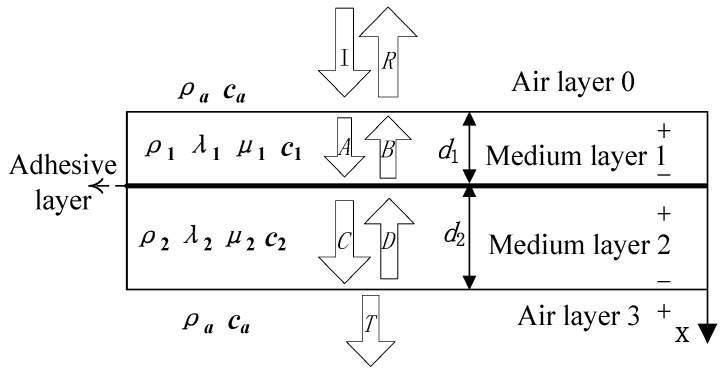
Schematic diagram of normal incidence air-coupled ultrasonic inspection.

Figure 1 shows the physical model for the detection of the kissing bond interface using normal incidence air-coupled ultrasound. The vertical-incidence method is adopted in the experiment. When the normally plane longitudinal wave is incident on the surface of the tested materials, parts of them are reflected from the air-solid interface and the others transmit into the tested materials. If the amplitude of the incident wave is seen as unit 1, *R* and *T*, the amplitude of the reflection and transmission, can be considered the reflection coefficient and transmission coefficient. *A* and *B* are the amplitude of incidence and reflection on the adhesive interface, respectively, and *C* and *D* are the amplitude on solid-air interface. The symbols + and − denote the top and bottom surfaces of the materials.

As shown in Figure 1, the tested material is composed of two plates which are homogeneous, isotropic, linear elastic materials. Medium layer 1 (density *ρ*_1_, thickness *d*_1_, Lame constants *λ*_1_ and *μ*_1_ and velocity *c*_1_) and medium layer 2 (density *ρ*_2_, thickness *d*_2_, Lame constants *λ*_2_ and *μ*_2_ and velocity *c*_2_) are held together by an adhesive layer. The property of the adhesive layer is negligible, which is substituted for the spring model proposed by Newmark, et al. [30]. The top and bottom layer are air, density and velocity of which are denoted *ρ_a_*, *c_a_*, respectively. *x* denotes the coordinate axis.

### 2.2. Mathematical Model

The location which the sound waves transmit into each layer interface is set as the coordinate system’s origin this layer. The sound pressure *p* and particle velocity *v* in every layer could be expressed as:

(1) $$p_{0}{=e}^{j\left({\omega t-k}_{0}x\right)}{+Re}^{j\left({\omega t+k}_{0}x\right)}$$

(2) $$p_{1}{=Ae}^{j\left({\omega t-k}_{1}x\right)}{+Be}^{j\left({\omega t+k}_{1}x\right)}$$

(3) $$p_{2}{=Ce}^{j\left({\omega t-k}_{2}x\right)}{+De}^{j\left({\omega t+k}_{2}x\right)}$$

(4) $$p_{3}{=Te}^{j\left({\omega t-k}_{3}x\right)}$$

(5) $$\upsilon_{0}=\frac{1}{z_{0}}{(e}^{j\left({\omega t-k}_{0}x\right)}{-Re}^{j\left({\omega t+k}_{0}x\right)})$$

(6) $$\upsilon_{1}=\frac{1}{z_{1}}{(Ae}^{j\left({\omega t-k}_{1}x\right)}{-Be}^{j\left({\omega t+k}_{1}x\right)})$$

(7) $$\upsilon_{2}=\frac{1}{z_{2}}{(Ce}^{j\left({\omega t-k}_{2}x\right)}{+De}^{j\left({\omega t+k}_{2}x\right)})$$

(8) $$\upsilon_{3}=\frac{1}{z_{3}}{(Te}^{j\left({\omega t-k}_{3}x\right)})$$

where *p* and *v* are the sound pressure and particle displacement speed, respectively. *ω* is the circular frequency. *k* = *ω*/*c*, where *k* is the wave number. *z* = *ρc*, where *z* is the material acoustic impedance. The subscript *m* (*m* = 0, 1, 2, 3) represents the corresponding layer parameters. j is the symbol of the imaginary part. Considering the upper and lower surfaces of the tested material are not adhesive, the sound pressure and particle displacement speed at the air-solid interface are continuous, and these boundary conditions are mathematically described as:

(9) $$\begin{array}{c} p_{0}^{-}{=p}_{1}^{+},\;\upsilon_{0}^{-}=\upsilon_{1}^{+}\\ p_{2}^{-}{=p}_{3}^{+},\;\upsilon_{2}^{-}=\upsilon_{3}^{+} \end{array}$$

Putting Equation (9) into matrix form can be formulated as:

(10) $$\left[\begin{array}{c} A\\ B \end{array}\right]=\left[\begin{array}{cc} 1/2 & z_{1}/2\\ 1/2 & {-z}_{1}/2 \end{array}\right]\left[\begin{array}{cc} 1 & 1\\ 1/z_{0} & -1/z_{0} \end{array}\right]\left[\begin{array}{c} 1\\ R \end{array}\right]$$

(11) $$\left[\begin{array}{c} T\\ T \end{array}\right]=\left[\begin{array}{cc} e^{{-jk}_{2}d_{2}} & e^{{jk}_{2}d_{2}}\\ \frac{z_{3}}{z_{2}}e^{{-jk}_{2}d_{2}} & -\frac{z_{3}}{z_{2}}e^{{jk}_{2}d_{2}} \end{array}\right]\left[\begin{array}{c} C\\ D \end{array}\right]$$

If the bonding of tested material shown in Figure 1 is imperfect, and the thickness of the layer is much smaller than the wavelength, the ultrasonic wave interaction with this interface can be described using spring boundary conditions:

(12) $$\frac{\partial p_{1}^{-}}{\partial t}=K(\upsilon_{1}^{-}-\upsilon_{2}^{+}),\;p_{1}^{-}=p_{2}^{+}$$

where *K* is the distributed spring constant per unit area (N·m^−3^). Putting Equation (12) into matrix form can be formulated as:

(13) $$\left[\begin{array}{c} C\\ D \end{array}\right]=\left[\begin{array}{cc} -\frac{z_{2}}{2K} & 1/2\\ \frac{z_{2}}{2K} & 1/2 \end{array}\right]\left[\begin{array}{cc} (j\omega -\frac{K}{z_{1}}{)e}^{{-jk}_{1}d_{1}} & (j\omega +\frac{K}{z_{1}}{)e}^{{jk}_{1}d_{1}}\\ e^{{-jk}_{1}d_{1}} & e^{{jk}_{1}d_{1}} \end{array}\right]\left[\begin{array}{c} A\\ B \end{array}\right]$$

By combining Equation (10), Equation (11), and Equation (13), the dependence of the transmission and reflection coefficients can be described as:

(14) $$\left[\begin{array}{c} T\\ T \end{array}\right]=N\left[\begin{array}{c} 1\\ R \end{array}\right]$$

where the matrix *N*, symbolized as *N* = $\left[\begin{array}{cc} n_{11} & n_{12}\\ n_{21} & n_{22} \end{array}\right]$, is equal to the matrix-chain multiplication from Equation (10), Equation (11), and Equation (13). The transmission coefficients can be described as:

(15) $$T=\frac{n_{11}n_{22}{-n}_{21}n_{12}}{n_{22}{-n}_{12}}$$

### 2.3. Analysis

Some special cases are considered. Firstly, if both *k*_1_*d*_1_ and *k*_2_*d*_2_ equal *n*π (*n* = 0, 1, 2…), namely the thickness of both plates are an integral multiple of the half wavelength at the same time. The transmission coefficient *T* reduces to:

(16) $$T=\frac{2}{\frac{{j\omega z}_{0}}{K}+2}$$

In terms of the previous works [31,32,33], the value of *K*, including the weakly-bonded or perfectly-bonded, taken into account in research process is much more than 10^12^ N·m^−3^. In these cases, the transmission coefficient *T* is approximate to 1.

Secondly, when the interface stiffness coefficient is infinite (*K*→+∞), the transmission coefficient in Equation (15) is given by the following equation:

(17) $$T=\frac{-2}{\left[2\cos(k_{1}d_{1})\cos(k_{2}d_{2})-(\frac{z_{1}}{z_{2}}+\frac{z_{2}}{z_{1}})\sin(k_{1}d_{1})\sin(k_{2}d_{2})]+i[(\frac{z_{0}}{z_{1}}+\frac{z_{1}}{z_{0}})\sin(k_{1}d_{1})\cos(k_{2}d_{2})+(\frac{z_{0}}{z_{2}}+\frac{z_{2}}{z_{0}})\cos(k_{1}d_{1})\sin(k_{2}d_{2}\right)}$$

If the specified values of the materials’ properties are not available, the extreme points of the frequency have no analytical solution from Equation (17). However, when both bonding material impedances are equal (*z*_1_ = *z*_2_), the transmission coefficient can reduce to:

(18) $$T=\frac{-2}{{2\cos(k}_{1}d_{1}{+k}_{2}d_{2})+i(\frac{z_{1}}{z_{0}}+\frac{z_{0}}{z_{1}}{)\sin(k}_{1}d_{1}{+k}_{2}d_{2})}$$

In this case, the extreme points are easily obtained, i.e., when *k*_1_*d*_1_ + *k*_2_*d*_2_ = *n*π (*n* = 0, 1, 2…), the transmission coefficient *T* reaches the extreme value.

## 3. Numerical Calculation

### 3.1. The Same Material and Equal Thickness 

The physical parameters of the materials, which include p-wave velocity, thickness, and density, have been measured by an experimental method and listed in Table 1. The p-wave velocity was measured by the time-difference method and the density was obtained by dividing mass by volume. The thickness can be measured by a Vernier caliper. The experimental results show that these properties are uniform in different places and different directions, so these materials are regarded as isotropic materials. 

**Figure 2:**
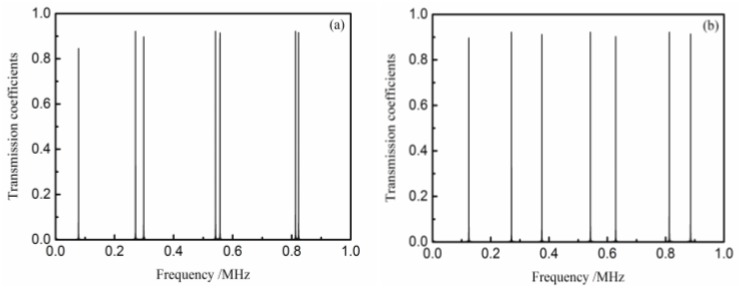

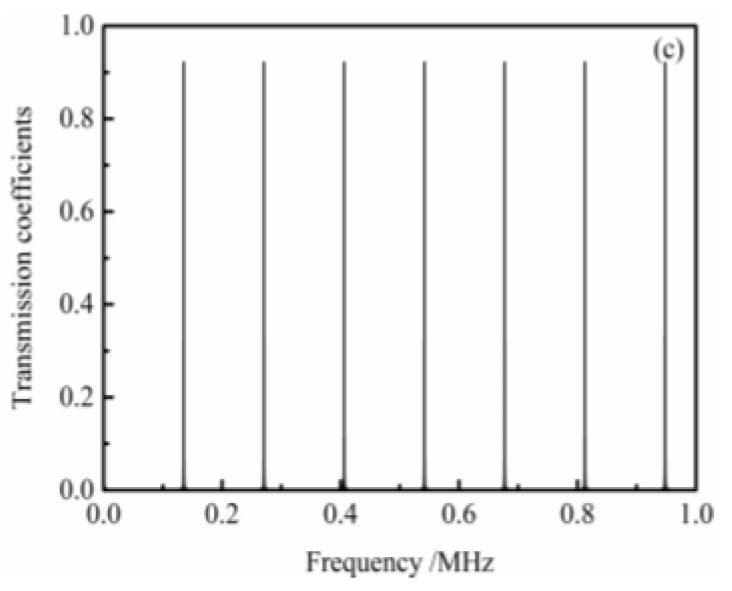
The transmission spectrum from equal-thickness PMMA plate (5 mm): (**a**) *K* = 10^12^ N·m^−3^; (**b**) *K* = 10^13^ N·m^−3^; and (**c**) *K* = 10^20^ N·m^−3^.

When both medium layers are polymethyl methacrylate (PMMA) and their thicknesses are 5 mm, the ultrasonic transmission spectrum can be obtained by calculating Equation (15) for different interfacial stiffnesses. Figure 2 shows the transmission spectrum from different interfacial stiffnesses between the two equal-thickness PMMA plates. If *kd* = *n*π (*n* = 0, 1, 2…), emerging on the spectrum, the transmission peaks cannot shift with the change of the interfacial stiffness. Other transmission peaks shift to a higher frequency area with the increase of the interfacial stiffness. If *K* = 10^20^ N·m^−3^ (perfect interface), the adhesive tested material can be viewed as an integral plate whose thickness equals the sum of two pieces of adhesive material, and the transmission peaks emerge at *kd* = *n*π/2. This result from the simulation is completely in accordance with the half-wave resonance theory. 

**Figure 3:**
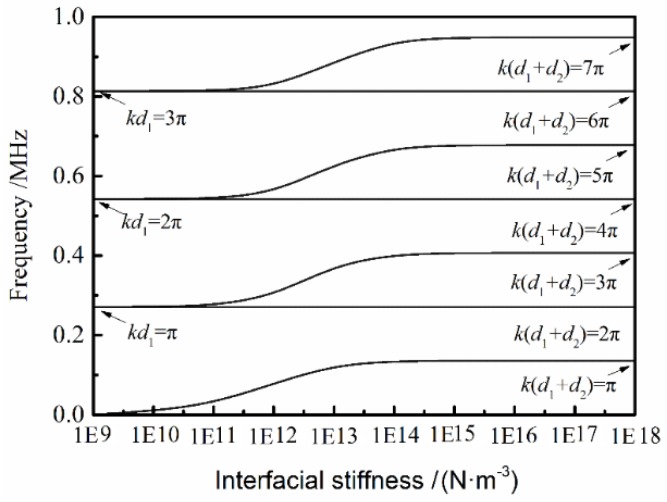
Transmission spectrum peaks as a function of the interfacial stiffness from the two equal-thickness (5 mm) PMMA plates.

Figure 3 shows the transmission peaks’ positions as a function of the interfacial stiffness, when the adhesive materials are both 5-mm-thick PMMA plates. It can be seen that as *K* < 10^11^ N·m^−3^, the peaks are at *kd* = *n*π (*n* = 0, 1, 2…), and these peaks do not change with the increase of the interfacial stiffness. When *K*→10^11^ N·m^−3^, others peaks appear nearby the original peaks, and the interfacial stiffness increase results in these peaks shift to a higher frequency, and as *K*→10^18^ N·m^−3^ these peaks shift to *kd* = *n*π/2 (*n* = 0, 1, 2…). In this case, the tested material is equivalent to a whole plate, and its thickness is equal to the sum of the two adhesive plates. 

It can also be observed that different frequencies have different sensitivities to the change of the interfacial stiffness from Figure 3. The lower frequencies in these movable frequency areas are more sensitive when the interfacial stiffness is lower. In other words, the higher-frequency peaks show a ‘later shift’ than lower-frequency peaks.

### 3.2. The Same Material and Unequal-Thickness

The transmission spectra of the ultrasound from a thin plate into a thick plate or from a thick plate into a thin plate are the same through the numerical calculation. The incident direction is not discussed in the following section.

**Figure 4:**
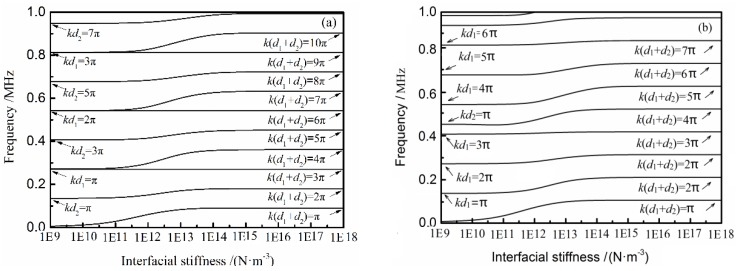
The dependence of frequency for transmission peaks on the interfacial stiffness for PMMA plates, (**a**) *d*_1_ = 5 mm, *d*_2_ = 10 mm; and (**b**) *d*_1_ = 10 mm, *d*_2_ = 3 mm.

Figure 4a shows the dependence of frequency for transmission peaks on the interfacial stiffness calculated from Equation (15), when the thickness of two PMMA plates are 10 mm and 5 mm, respectively, and Figure 4b illustrates that the thicknesses of double layers are 10 mm and 3 mm, respectively. As shown in Figure 4a, when *K*→10^18^ N·m^−3^ (perfectly bonded), the transmission peaks appear at *k* (*d*_1_ + *d*_2_) = *n*π (*n* = 1, 2, 3…). In this case, it is also in good agreement with the half-wave resonance theory. One can also see that as *K* < 10^11^ N·m^−3^ (weak bonded), the transmission peaks are at *kd*_1_ = *n*π or *kd*_2_ = *n*π (*n* = 0, 1, 2…). With the increase of the interfacial stiffness, these peaks shift to the higher-frequency area. The condition when *kd*_1_ = *n*π or *kd*_2_ = *n*π can be seen as the starting points that the transmission peaks move toward the higher frequency and the condition when *k* (*d*_1_ + *d*_2_) = *n*π as the stopping points.

These regulars which are described in Figure 4a are also suitable for Figure 4b. There is the phenomenon of ‘later shift’ of higher frequencies in both figures. However, not all the peaks have the same sensibility. For example, in Figure 4b, the peak that when the starting point is *kd*_1_ = 3π obviously have less sensibility than the one that when the starting point is *kd*_1_ = 4π.

### 3.3. The Unequal-Thickness Materials

**Figure 5:**
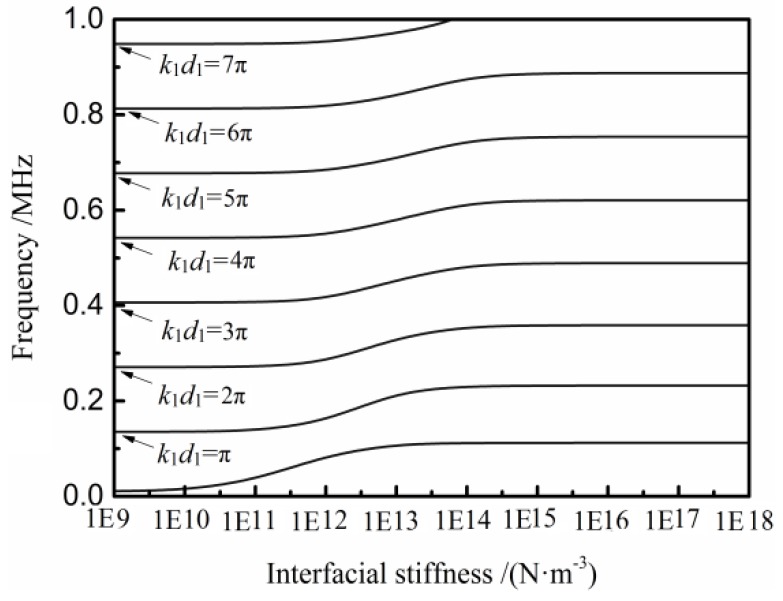
The dependence of the frequency for transmission coefficient peaks on the interfacial stiffness: the 1-mm-thick aluminum plate bonded to the 10-mm-thick PMMA plate.

Figure 5 shows the relationship between the frequency for the transmission coefficient peaks and the interface stiffness, as the tested material is the 1-mm-thick aluminum plate bonded to the 10-mm-thick PMMA plate. From Figure 5, the phenomenon of ‘later shift’ is also observed and the sensitivity of the different transmission peaks is not the same. Meanwhile, the move starting points of the transmission peaks are in the condition when *k*_1_*d*_1_ = *n*π (*n* = 0, 1, 2…). However, the stopping point could not be described as an algebraic formula, because it is related to the substrate material parameters, such as the density and the ultrasound velocity, etc., in addition to the wave numbers and plate thickness. 

## 4. Experimental 

### 4.1. Experimental Apparatus

**Figure 6:**
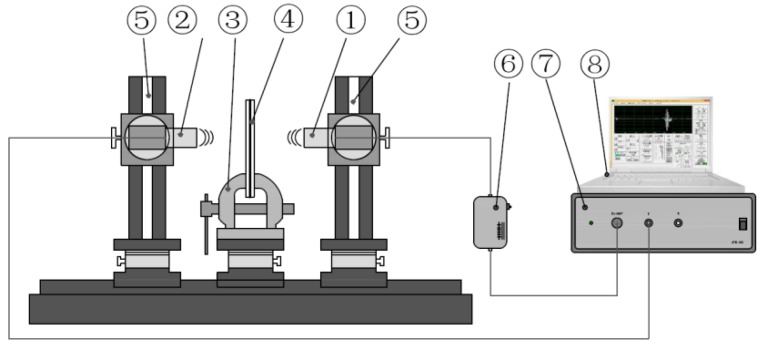
Schematic diagram of the experimental setup.

The ultrasonic system employed to research on the transmission characteristics of the air-coupled ultrasound is illustrated in Figure 6. ① and ② are, respectively, the transmitting transducer and the receiving transducer, which are provided by Japan Probe Co. Ltd. (Yokohama, Kanagawa Prefecture, Japan) in Japan. These transducers, whose central lines are coincident and horizontal, are fixed on opposite sides of the tested material ④. According to theoretical analysis and the measurements performed by An et al. [32], the transducers with a central frequency of 800 kHz and a 7 mm × 10 mm rectangle active element were chosen in the experiment. The distance between the transmitting transducer and the tested material is 40 mm, and the distance between the receiving transducer and the tested material is 20 mm, which are both in the near-field zone of the ultrasonic transducer. These distance parameters are provided by the transducer supplier.

The sample of the tested material is installed perpendicular to the central line of the transducers. ③ is a U-shaped retaining clip, which is used to fix the tested material. ⑤ is the fixation tool of the transducers. ⑥ and ⑦ are the receiver power amplifier and the ultrasonic pulser/receiver, respectively. ⑧ is a personal computer (PC) used to control ⑦ and analyze experimental data. 

Ultrasonic pulses generated from the ultrasonic pulser/receiver propagate through the transmitting transducer, the air and the tested specimen and finally are received by the receiving transducer. The received signals are amplified by receiving power amplifier which flat gain is 60 dB, and then transmitted in turn to the ultrasonic pulser/receiver, and the personal computer. In this experiment, the sampling frequency is 10 MHz, and the transmit voltage of the ultrasonic pulser is 600 V. The time domain signal is easy to transform into the frequency domain using Fast Fourier Transform (FFT). 

### 4.2. Specimen Preparation

Using ultrasonic c-scan method, all substrates are estimated to ensure that there are no gross imperfections and anomalies. They are cut into the size of 60 mm × 60 mm, the clutter and burr of which are cleared to reduce measurement error. The material parameters are listed in Table 1.

Two substrates are bonded by taking a two-part epoxy adhesive, which consist of A epoxy adhesive and B epoxy adhesive with the same proportion and stirred uniformly. The bond property of the epoxy adhesive can be influenced by the stressed force, humidity, and temperature, so these conditions have almost been maintained consistently during the experiment. In terms of the instruction of the two-part epoxy adhesive, the bonding strength will increase with the increase of the bonding time and achieve the maximum value after 10 h under the usual circumstances. Consequently, the bonding specimens should be measured when the bonding time is 1, 5, and 10 h, respectively. Before the cohesive bodies are measured, they are pressured by heavy stuff for eliminating bubbles between two plates. The following equation is used to give the thickness of the adhesive layer:

(19) $$t_{\mathrm{adhesive}}=t_{\mathrm{total}}-t_{\mathrm{sum}}$$

where *t*_adhesive_ is the thickness of the adhesive layer, *t*_total_ and *t*_sum_ are the whole thickness of the bonding body and the sum of the two substrates’ thicknesses, respectively, which can be measured by a Vernier caliper. *t*_adhesive_ is approx. 0.1 mm, so that its thickness and attenuation are neglected. 

### 4.3. Experimental Procedures

**Figure 7:**
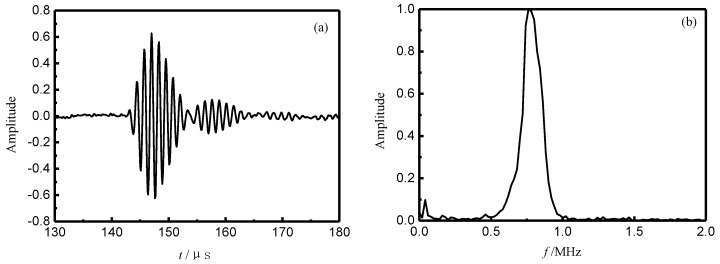
Time-domain waveform and frequency domain spectrum of the reference signal. (**a**) Time signals; and (**b**) frequency spectrum.

According to the theory analysis, the bonding quality can impact the transmission coefficient of the bonding structure. The transmission coefficient is used to indicate the bonding quality. For demonstrating the previous mentioned theory, the transmission coefficient should be measured in an experiment. The transmission coefficient can be expressed by the following equation [34]:

(20) $$T=\frac{O(f)}{I(f)}$$

where *O*(*f*) is the frequency spectrum of the transmission signal, and *I*(*f*) is the frequency spectrum of the incident signal. Firstly, when there is no bonding specimen between the transmitting transducer and the receiving transducer. Meanwhile, the receiving power amplifier, only for amplifying the amplitude of receive signal, is uninstalled. The time signals could be got and shown in Figure 7a. Secondly, a frequency spectrum can be calculated by FFT on the time signal. Finally, *I*(*f*) is the frequency spectrum of normalization and shown in Figure 7b. In addition, it is important to note that the normalization and uninstalling the amplifier could not influence the peak position of the transmission coefficient. 

The three types of bonding bodies are made by the above method, which included the same material and the same thickness (5 mm PMMA plate and 5 mm PMMA plate bonding), the same material and different thickness (10 mm PMMA plate and 5 mm PMMA plate bonding, 10 mm PMMA plate and 3 mm PMMA plate bonding), and different material and different thickness (10 mm PMMA plate and 1 mm aluminum plate bonding). According to the method shown in Figure 6 to assemble the equipment, some transmission signals could be recorded by measuring these materials. These signals transform into the frequency spectrum (*O*(*f*)) by FFT.

**Figure 8:**
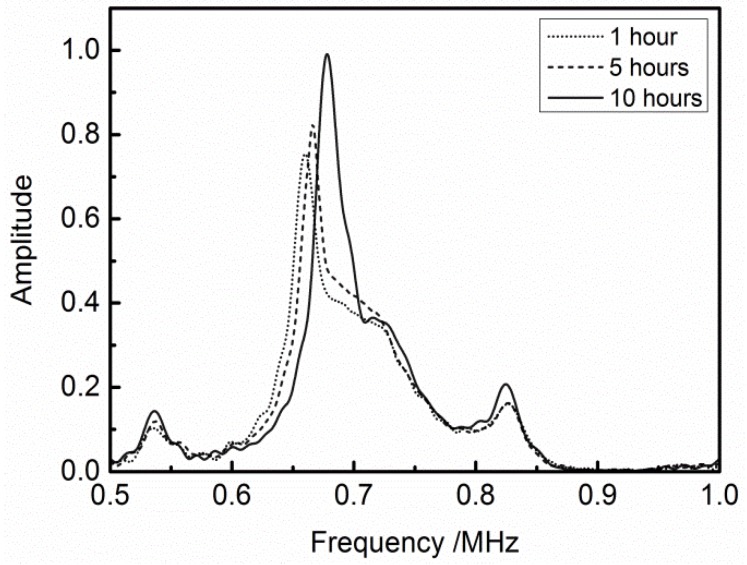
Shift in the transmission peaks due to variation of bonding time: two equal-thickness (5 mm) PMMA plates.

Figure 8 shows the frequency spectrums from measuring the adhesive bonding structure of equal-thickness (5 mm) PMMA plates. Comparison with the spectrums on the condition of the different bonding time, the middle peak shift towards the higher frequency area with the increase of the bonding time, but the peaks on both sides hardly move. Since the transmission signal amplified by the receiver power amplifier only represent the relative amount, the voltage amplitude which is from the receiver transducer is normalized. This phenomenon is almost consistent with previous theoretical analysis shown in Figure 3. The both side transmission peaks do not match together completely, which is caused by the bonding layer. The amplitude of the transmission peaks, which is not in the main band range, is small, but it also has a reference significance.

**Figure 9:**
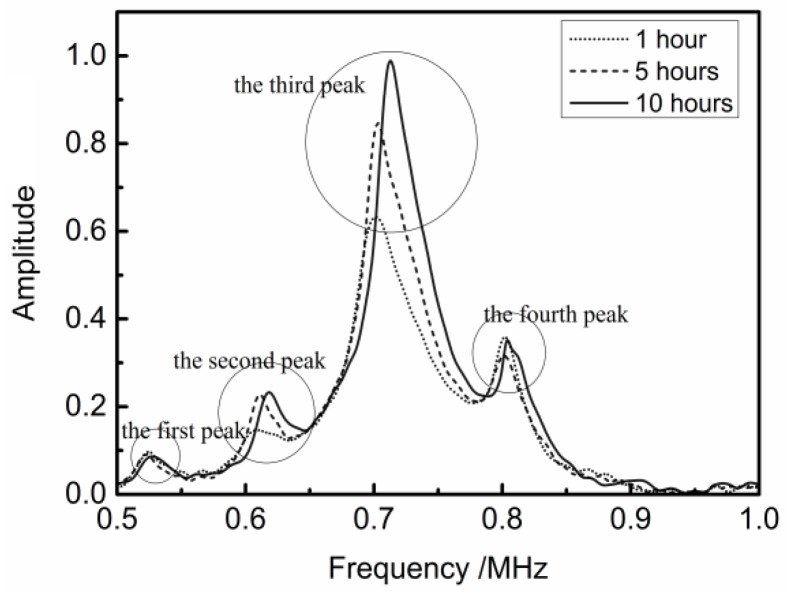
Shift in the transmission peaks due to the variation of the bonding time: 10 mm PMMA plate and 5 mm PMMA plate bonding.

Measuring the adhesive bonding structure of 10 mm-thickness and 5 mm-thickness PMMA plates, the frequency spectra are shown in Figure 9. The transmission peaks on both sides do not change and the two peaks in the middle shift towards the higher frequency area with the growing bonding time. Table 2 shows the peak position of the transmission coefficient at different bonding time which are shown in Figure 9. Through contrasting the second peak and the third peak in Table 2, the shift distance of the second peak is greater than the third one when the bonding time increases from 1 h to 5 h. However, when the bonding time increases from 5 h to 10 h, the third one is more sensitive than the second one. This phenomenon is agreement with the law of ‘later shift’ as mentioned above. 

**Figure 10:**
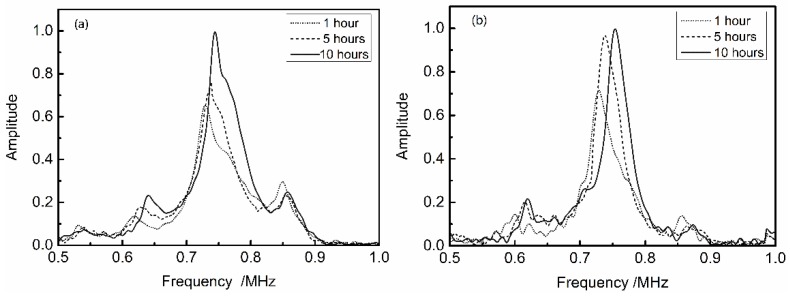
Shift in the transmission peaks due to variation of bonding time: (**a**) 10 mm PMMA plate and 3 mm PMMA plate bonding; and (**b**) 10 mm PMMA plate and 1 mm aluminum plate bonding.

Likewise, Figure 10 shows the frequency spectrum of two remaining situations, namely 10 mm PMMA plate and 3 mm PMMA plate bonding, 10 mm PMMA plate and 1 mm aluminum plate bonding. It can be seen that almost all transmission peaks shift towards the higher frequency area, and there is still the phenomenon of ‘later shift’. However, not every peak has the same sensitive to the bonding time. As shown in Figure 10a, the peak between 0.6 MHz and 0.7 MHz is the most sensitive compared with the other frequency area. Similarly, the peak between 0.7 MHz and 0.8 MHz is the most sensitive in Figure 10b.

## 5. Conclusions

The physical and mathematical models, which are used to describe the propagating characteristic of the air-ultrasound in double-layered adhesively-bonded structures, is established by the transfer matrix method. This mathematical model is simplified in some special cases. By analyzing this model, two laws have been presented. One is that when the thicknesses of both substrates are equal to an integral multiple of a half wavelength, the transmission coefficient *T* is approximate to one. This rule could be considered as an expansion of the half-wave theory. The other is that as the impedance of both substrates are equal and the interfacial stiffness is infinite, the transmission coefficient *T* reaches the extreme value when the sum thickness of both substrates is equal to half a wavelength.

The transmission spectra of the adhesive structures, which are made of different substrates, are presented by numerical calculation in the different interfacial stiffness. The calculation results show that the spectrum shifts toward higher frequencies with the increase of the interfacial stiffness, but the speed of moving varies in terms of the different interfacial stiffness. Some transmission peaks are relevant to the change of the interfacial stiffness, however, some are not sensitive, and even irrelevant, to it. Indeed, it is also shown that the higher frequency peaks are shifted later than the lower frequency peaks. The starting points that the transmission peaks move toward the higher frequency are located at *kd*_1_ = *n*π or *kd*_2_ = *n*π regardless of the property of the material. The stopping points, however, depend on the property of material, When two substrates are of the same material, the stopping points are located at *k* (*d*_1_ + *d*_2_) = *n*π, When two substrates are not of the same material, the stopping points could not be described by an algebraic formula. The phenomenon from experiments is in agreement with the calculation results above. In addition, all of the rules proved by experimental investigations provide the theoretical foundation for choosing the air-coupled transducer with optimal parameters, including the central frequency and its band to accurately evaluate the integrity of the adhesive structure.

## Figures and Tables

**Table 1:** Physical parameters of the materials.

Material	P-Wave Velocity (m/s)	Density (kg/m^3^)	Impedance (Rayl)	Thickness (mm)
Air	340	1.29	438.6	N/A
Polymethyl Methacrylate	2710	1200	3.252 × 10^6^	3, 5 and 10
Aluminum	6320	2770	17.5064 × 10^6^	1

**Table 2:** Peaks position at different bonding time.

Serial Number	Peak Position at 1 h (MHz)	Peak Position at 5 h (MHz)	Peak Position at 10 h (MHz)
The first peak	0.523	0.523	0.523
The second peak	0.607	0.613	0.620
The third peak	0.700	0.703	0.713
The fourth peak	0.803	0.803	0.806
